# Effect of radiation after surgery on the prognosis of children with Wilms tumor

**DOI:** 10.1371/journal.pone.0308824

**Published:** 2024-09-19

**Authors:** Songqiang Chen, Zhisheng Wan, Shaohua Hu, Weizhen Bu, Yiqun Lu, Zhenli Zhao

**Affiliations:** 1 Department of Urology, Hainan Women and Children’s Medical Center, Haikou, Hainan, China; 2 Department of Urology, Children’s Hospital of Fudan University, Shanghai, China; CNR, ITALY

## Abstract

**Background:**

To explore the association between radiation after surgery and the 5-year overall survival (OS) and 5-year cancer-specific survival (CSS) in patients with Wilms tumor.

**Methods:**

In this cohort study, 1564 participants were identified from the Surveillance, Epidemiology, and End Results (SEER) database. The univariate and multivariable COX proportional risk model as well as competitive risk model were used to explore the covariates associated with 5-year OS and 5-year CSS of patients with Wilms tumor and the correlation between radiation after surgery and 5-year OS or 5-year CSS of patients with Wilms tumor, respectively. The Kaplan-Meier curves of participants were plotted.

**Results:**

The median follow-up was 126.00 (84.00, 178.00) months. Patients receiving surgery had higher 5-year survival probability than those not receiving surgery, while participants receiving radiation after surgery showed poor 5-year survival than those not. After adjusting for covariates including age and SEER stage, increased risk of 5-year overall mortality in patients with Wilms tumor [hazard ratio (HR) = 1.62, 95% confidence interval (CI): 1.10–2.41). After the adjustment for confounding factors including age, SEER stage and ethnicity, increased risk of 5-year cancer-specific mortality of patients with Wilms tumor was observed in those receiving radiation after surgery (HR = 1.77, 95%CI: 1.13–2.79).

**Conclusion:**

Radiation after surgery was associated with poor prognosis of patients with Wilms tumor, which indicated that the clinicians should assess whether the patient was suitable for using radiation after surgery.

## Introduction

Wilms tumor, namely nephroblastoma, is one of the most prevalent embryonal tumors in children younger than five years old [1]. Wilms tumor was thought to arise from nephrogenic rests, the remnants of embryonal development leading to malignant [2]. Wilms tumor accounts for nearly 95% of childhood kidney tumors, and the incidence of nephroblastoma in infants is about 1/1,000,000 [3]. At present, the survival rate of children with Wilms tumor can exceeded 90% in high-income countries based on modern multidisciplinary comprehensive treatments including surgery and chemotherapy [4]. The survival rates are still low in low-income regions [5,6]. To identify more reliable factors to improve the outcomes of Wilms tumor was of great value in the management of these patients.

Currently, surgery is the standard treatment for Wilms tumor [7]. The National Comprehensive Cancer Network (NCCN) suggested that adjuvant radiotherapy can be used for children with Wilms tumor at high post-surgery risk, but not for low-risk children in early stage disease [8]. There was evidence revealed that radiotherapy might have adverse effects on normal tissues, which may lead to growth and development delay in children or increased risk of gastrointestinal, lung or heart dysfunction, and second primary cancer (SPM) [9,10]. Previous studies indicated that the 30-year cumulative incidence of SPM in children with cancer who survived more than 5 years was 20.5%, which was six times than the general population, and children who received radiotherapy had a higher risk of SPM [11,12]. A recent study analyzing factors affecting the survival of children with Wilms tumor depicted that radiotherapy did not significantly improve overall survival of these patients [13]. To explore the influence of radiation after surgery on the prognosis of children with Wilms tumor and analyze the applicability of radiotherapy for children with Wilms tumor receiving surgical treatment are necessary for the management of these patients.

In this study, we explored the association between radiation after surgery and the 5-year overall survival (OS) rate and 5-year cancer-specific survival (CSS) rate in children and adolescents with Wilms tumor based on the data from the Surveillance, Epidemiology, and End Results (SEER) database. Subgroup analysis was stratified by age, gender, SEER stage, laterality, surgery type, and chemotherapy.

## Methods

### Study design and population

In this cohort study, 2172 participants who were diagnosed with primary Wilms tumor between 2000–2015 were identified in the SEER 18 database, Nov 2020 Sub (2000–2018) (https://seer.cancer.gov/seerstat/). The SEER*Stat 8.4.0 software was used to generate case lists and obtain corresponding data. The SEER database draws from 21 population-based cancer registries to provide information on approximately 28% of the US population, which holds annually uploaded data on patient demographics, tumor pathology, anatomic sites of the tumor, stage at diagnosis, first course of treatment modalities, and follow-up vital status [14]. The pathologically confirmation of primary Wilms tumor was screened from SEER database based on ICD-O-3 with histological code of 8960/3. Patients who aged ≥20 years, subjects not receiving surgical treatment, participants receiving radiotherapy before or during surgery, those not knowing whether radiotherapy after surgery, and people without complete survival data were excluded. Finally, 1564 participants were included. The requirement of ethical approval for this was waived by the Institutional Review Board of Hainan Women and Children’s Medical Center, because the data was accessed from SEER (a publicly available database). The need for written informed consent was waived by the Institutional Review Board of Hainan Women and Children’s Medical Center due to retrospective nature of the study.

### Potential covariates

Age (years), gender (female or male), ethnicity (White, Black, other or unknown), laterality (unilateral left-sided, unilateral right-sided, unilateral but unknown side, or bilateral), tumor size (≤7cm, 7-10cm, >10cm or unknown), SEER stage (distant, non-distant or unknown), chemotherapy (no/unknown or yes), surgery [nephron sparing surgery (NSS), radical nephrectomy (RN) or surgery-not otherwise specified (NOS)] and SPM (no or yes) were potential covariates analyzed in this study.

SEER stage was defined based on SEER Combined Summary Stage 2000 (2004–2017), which showed that the mortality rates of regional and local were low, and they were combined into non-distant metastasis. The surgical methods were mainly divided into NSS and RN. According to the code of surgery of primary site, NSS includes 10–30, RN includes 40/50. 70–90 indicates surgery [Not Otherwise Specified (NOS)].

### Main and outcome variables

Radiotherapy was the main variable in our study. Whether radiotherapy was performed after surgery was identified based on the variable *RX Summ Surgery/Radiation Sequence (RX Summ—Surg/Rad Seq)* in the SEER database, which indicated the order of radiotherapy and surgery. 0 (no radiation and/or no surgery; unknown if surgery and/or radiation given), 2 (radiation before surgery), 3 (radiation after surgery), 4 (radiation both before and after surgery), 5 (intraoperative radiation), 6 (intraoperative radiation with other radiation given before and/or after surgery), 7 (surgery both before and after radiation), and 9 (sequence unknown, but both surgery and radiation were given).

The 5-year OS was identified based on the variable of Vital status recode (study cutoff used) in the SEER database and the 5-year CSS Specific survival at 5-year CSS was identified based on cause-specific death classification, SEER other cause of death classification combined with variable Vital status recode (study cutoff used). The median follow-up was 126.00 (84.00, 178.00) months.

### Statistical analysis

The normal-distributed measurement data was shown by mean ± standard error (SD) and non-normal distributed measurement data were expressed as the median and quartiles [M (Q_1_, Q_3_)]. The enumeration data were described by the number of cases and percentages [n (%)]. The comparison of measurement data involving normal distribution data was conducted by t test. Non-normal-distributed measurement data were compared by Mann-Whitney U rank sum test. The enumeration data were compared by chi-square test or Fisher’s exact probability method. Missing data were dealt with multiple imputation, and sensitivity analysis was performed to compare the results before and after missing data imputation. The univariate and multivariable COX proportional risk model was used to explore the covariates associated with 5-year OS of patients with Wilms tumor and the correlation between radiation after surgery and 5-year OS of patients with Wilms tumor. The univariate and multivariable competitive risk model was used to explore the covariates associated with the 5-year CSS and the association between radiation after surgery and 5-year CSS of patients with Wilms tumor. Subgroup analysis were stratified according to age, gender, SEER stage, laterality surgery type, and chemotherapy. The Kaplan-Meier curves of participants were plotted. Hazard ratio (HR) and 95% confidence interval (CI) were applied as effect size. SAS9.4 software (SAS Institute Inc., Cary, NC, USA) was used for data extraction, and statistical analysis.

## Results

### Comparisons between participants receiving radiation after surgery or not

**Fig 1:**
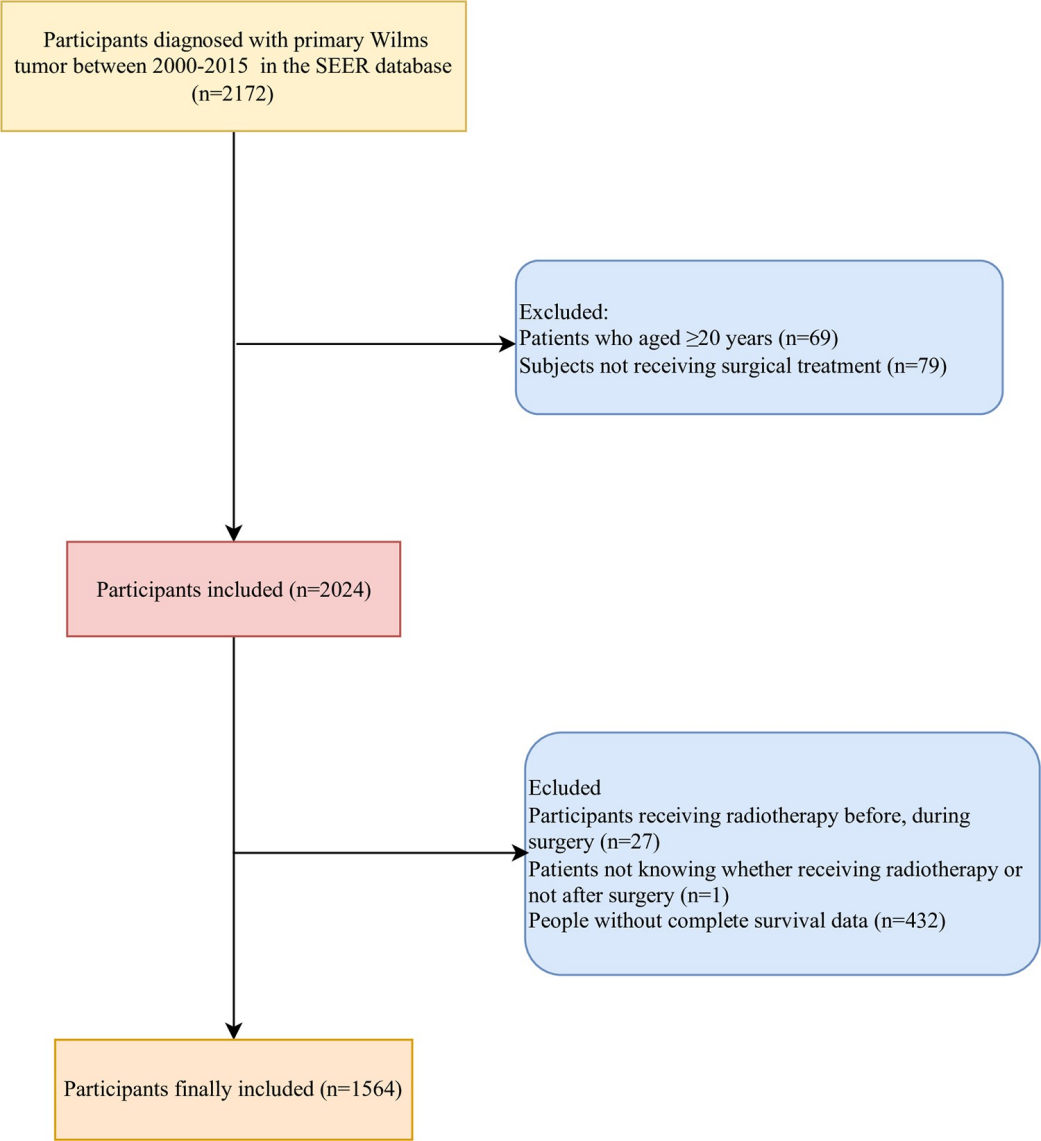
The screen process of the participants.

In total, the data of 2172 participants diagnosed with primary Wilms tumor was collected from the SEER database. Among them, patients who aged ≥20 years (n = 69), subjects not receiving surgical treatment (n = 79), participants receiving radiotherapy before, during surgery (n = 27) or those not knowing whether receiving radiotherapy or not after surgery (n = 1), and people without complete survival data (n = 432) were excluded. Finally, 1564 participants were included. The screen process was exhibited in Fig 1.

Compared with patients not receiving radiation after surgery, the median age of patients receiving radiation after surgery was higher (2.00 years vs 4.00 years). The percentages of participants with different laterality, tumor size, and SEER stage were statistically different between participants receiving radiation after surgery or not. The percentage of subjects receiving chemotherapy in the radiation after surgery group was higher than the non-radiation after surgery group (96.76% vs 86.78%). More detailed information of participants receiving radiation after surgery or not was presented in Table 1.

**Table 1:** The baseline characteristics of participants.

Variables	Total (n = 1564)	Radiation after surgery (n = 709)	No radiation after surgery (n = 855)	Statistics	*P*
Age, years, M (Q_1_, Q_3_)	3.00 (1.00, 5.00)	4.00 (2.00, 5.00)	2.00 (1.00, 4.00)	Z = 11.630	<0.001
Sex, n (%)				χ^2^ = 3.564	0.059
Female	826 (52.81)	393 (55.43)	433 (50.64)		
Male	738 (47.19)	316 (44.57)	422 (49.36)		
Ethnicity, n (%)				χ^2^ = 2.019	0.568
White	1174 (75.06)	537 (75.74)	637 (74.50)		
Black	286 (18.29)	130 (18.34)	156 (18.25)		
Other	90 (5.75)	38 (5.36)	52 (6.08)		
Unknown	14 (0.90)	4 (0.56)	10 (1.17)		
Laterality, n (%)				-	0.032
Unilateral left-sided	733 (46.87)	350 (49.37)	383 (44.80)		
Unilateral right-sided	723 (46.23)	323 (45.56)	400 (46.78)		
Unilateral but unknown side	5 (0.32)	2 (0.28)	3 (0.35)		
Bilateral	103 (6.59)	34 (4.80)	69 (8.07)		
Tumor size, n (%)				χ^2^ = 37.330	<0.001
≤7cm	235 (15.03)	83 (11.71)	152 (17.78)		
7-10cm	243 (15.54)	97 (13.68)	146 (17.08)		
>10cm	584 (37.34)	321 (45.28)	263 (30.76)		
Unknown	502 (32.10)	208 (29.34)	294 (34.39)		
SEER stage, n (%)				χ^2^ = 150.526	<0.001
Distant	282 (18.03)	220 (31.03)	62 (7.25)		
Non-distant	815 (52.11)	298 (42.03)	517 (60.47)		
Unknown	467 (29.86)	191 (26.94)	276 (32.28)		
Chemotherapy, n (%)				χ^2^ = 48.549	<0.001
No/Unknown	136 (8.70)	23 (3.24)	113 (13.22)		
Yes	1428 (91.30)	686 (96.76)	742 (86.78)		
Surgery, n (%)				χ^2^ = 9.716	0.008
NSS	111 (7.10)	40 (5.64)	71 (8.30)		
RN	1340 (85.68)	605 (85.33)	735 (85.96)		
Surgery (NOS)	113 (7.23)	64 (9.03)	49 (5.73)		
SPM, n (%)				-	0.076
No	1553 (99.30)	701 (98.87)	852 (99.65)		
Yes	11 (0.70)	8 (1.13)	3 (0.35)		
5-year mortality, n (%)				χ^2^ = 23.596	<0.001
Alive	1409 (90.09)	613 (86.46)	796 (93.10)		
Dead of this cancer	135 (8.63)	88 (12.41)	47 (5.50)		
Dead of other disease	20 (1.28)	8 (1.13)	12 (1.40)		
Survival time, months, M (Q_1_, Q_3_)	126.00 (84.50, 178.00)	117.00 (76.00, 166.00)	135.00 (90.00, 184.00)	Z = -4.163	<0.001

M: Median, Q_1_:1st Quartile, Q_3_:3rd Quartile, SEER: Surveillance, Epidemiology, and End Results, NSS: Nephron-sparing surgery, RN: Radical nephrectomy, NOS: Not otherwise specified, SPM: Second primary malignancy.

### Potential confounding factors for the 5-year OS or 5-year CSS of patients with Wilms tumor

The univariate COX proportional risk model was used to explore the covariates associated with 5-year OS of patients with Wilms tumor and the univariate competitive risk model was used to explore the covariates associated with the 5-year CSS of patients with Wilms tumor. As shown in Table 2, age (HR = 1.09, 95%CI: 1.05–1.14), and non-distant SEER stage (HR = 0.26, 95%CI: 0.17–0.39) were confounding factors associated with the 5-year OS of patients with Wilms tumor. Age (HR = 1.11, 95%CI: 1.07–1.15), ethnicity, and non-distant SEER stage (HR = 0.35, 95%CI: 0.22–0.55) were confounding factors for the 5-year OS or 5-year CSS of patients with Wilms tumor (Table 2).

**Table 2:** Potential confounding factors for the 5-year OS or 5-year CSS of patients with Wilms tumor.

Variables	OS		CSS	
	HR (95%CI)	*P*	HR (95%CI)	*P*
Age	1.09 (1.05–1.14)	<0.001	1.11 (1.07–1.15)	<0.001
Sex				
Female	Ref		Ref	
Male	1.01 (0.71–1.43)	0.964	1.02 (0.71–1.46)	0.928
Ethnicity				
White	Ref		Ref	
Black	1.11 (0.71–1.72)	0.651	1.03 (0.64–1.64)	0.910
Other	1.14 (0.55–2.34)	0.727	0.89 (0.39–2.03)	0.775
Unknown	0.93 (0.13–6.64)	0.939	0.00 (0.00–0.00)	<0.001
Laterality				
Unilateral left-sided	Ref		Ref	
Unilateral right-sided	0.79 (0.54–1.14)	0.200	0.75 (0.51, 1.09)	0.131
Unilateral but unknown side	2.70 (0.37–19.44)	0.325	2.75 (0.32, 23.96)	0.359
Bilateral	1.36 (0.73–2.52)	0.328	1.39 (0.75, 2.57)	0.299
Tumor size				
≤7cm	Ref		Ref	
7-10cm	0.69 (0.34–1.40)	0.304	0.89 (0.42–1.89)	0.767
>10cm	1.30 (0.77–2.21)	0.325	1.54 (0.86–2.77)	0.150
Unknown	0.99 (0.57–1.74)	0.980	1.22 (0.66–2.26)	0.533
Seer stage				
Distant	Ref		Ref	
Non-distant	0.26 (0.17–0.39)	<0.001	0.22 (0.15–0.34)	<0.001
Unknown	0.36 (0.23–0.56)	<0.001	0.35 (0.22–0.55)	<0.001
Chemotherapy				
No/Unknown	Ref		Ref	
Yes	1.66 (0.77–3.56)	0.193	1.50 (0.70–3.22)	0.298
Surgery				
NSS	Ref		Ref	
RN	1.28 (0.59–2.74)	0.531	1.14 (0.53–2.47)	0.737
Surgery (NOS)	1.73 (0.68–4.39)	0.251	1.73 (0.67–4.42)	0.255
SPM				
No	Ref		Ref	
Yes	2.19 (0.54–8.83)	0.272	1.18 (0.18–7.73)	0.861

Ref: Reference, HR: Hazard ratio, CI: Confidence interval, NSS: Nephron-sparing surgery, RN: Radical nephrectomy, NOS: Not otherwise specified, SPM: Second primary malignancy; OS: Overall survival, CSS: Cancer‐specific survival.

### Associations of radiation after surgery with 5-year OS and 5-year CSS of patients with Wilms tumor

**Fig 2:**
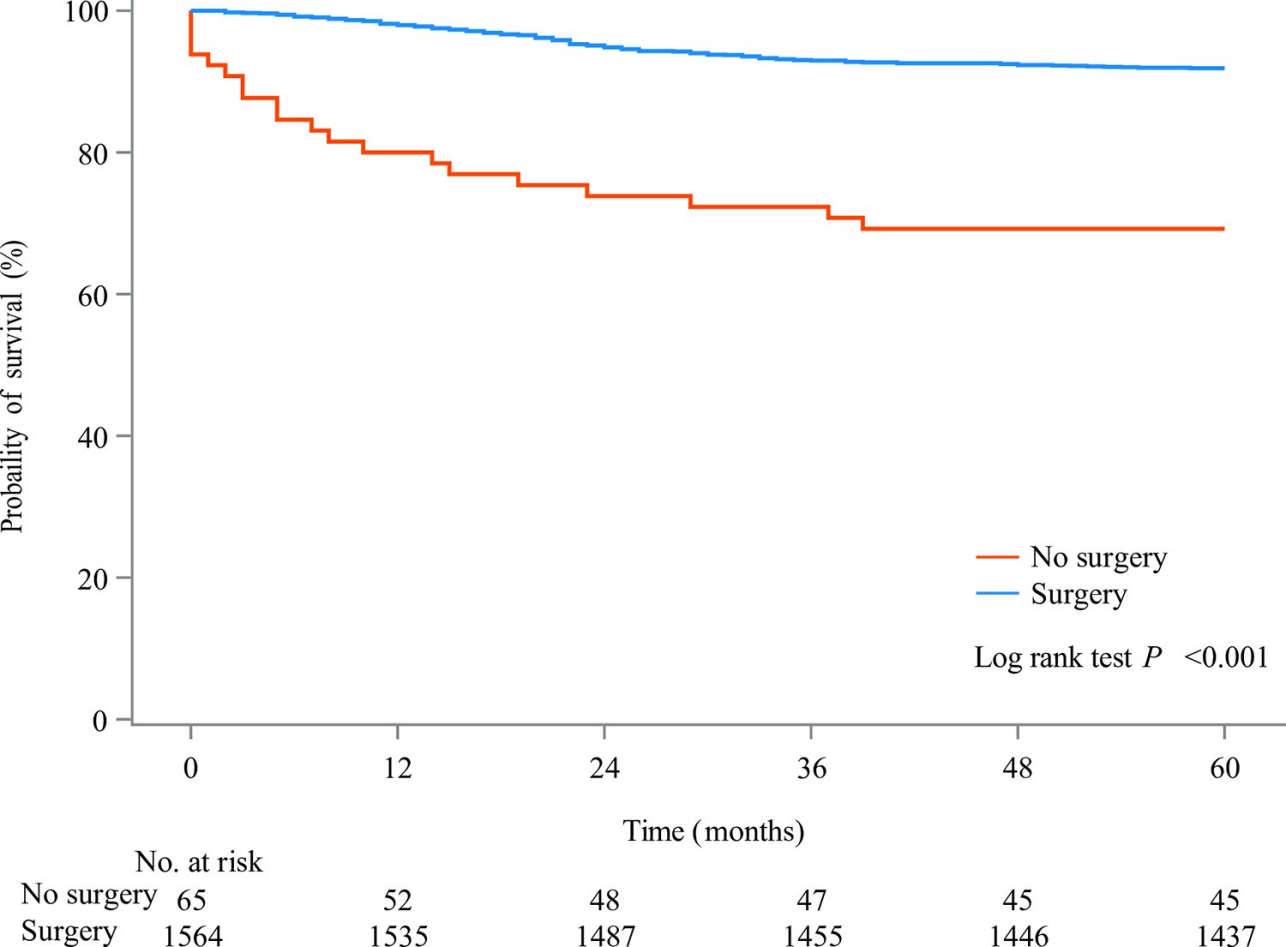
The KM curves of subjects receiving surgery or not.

**Fig 3:**
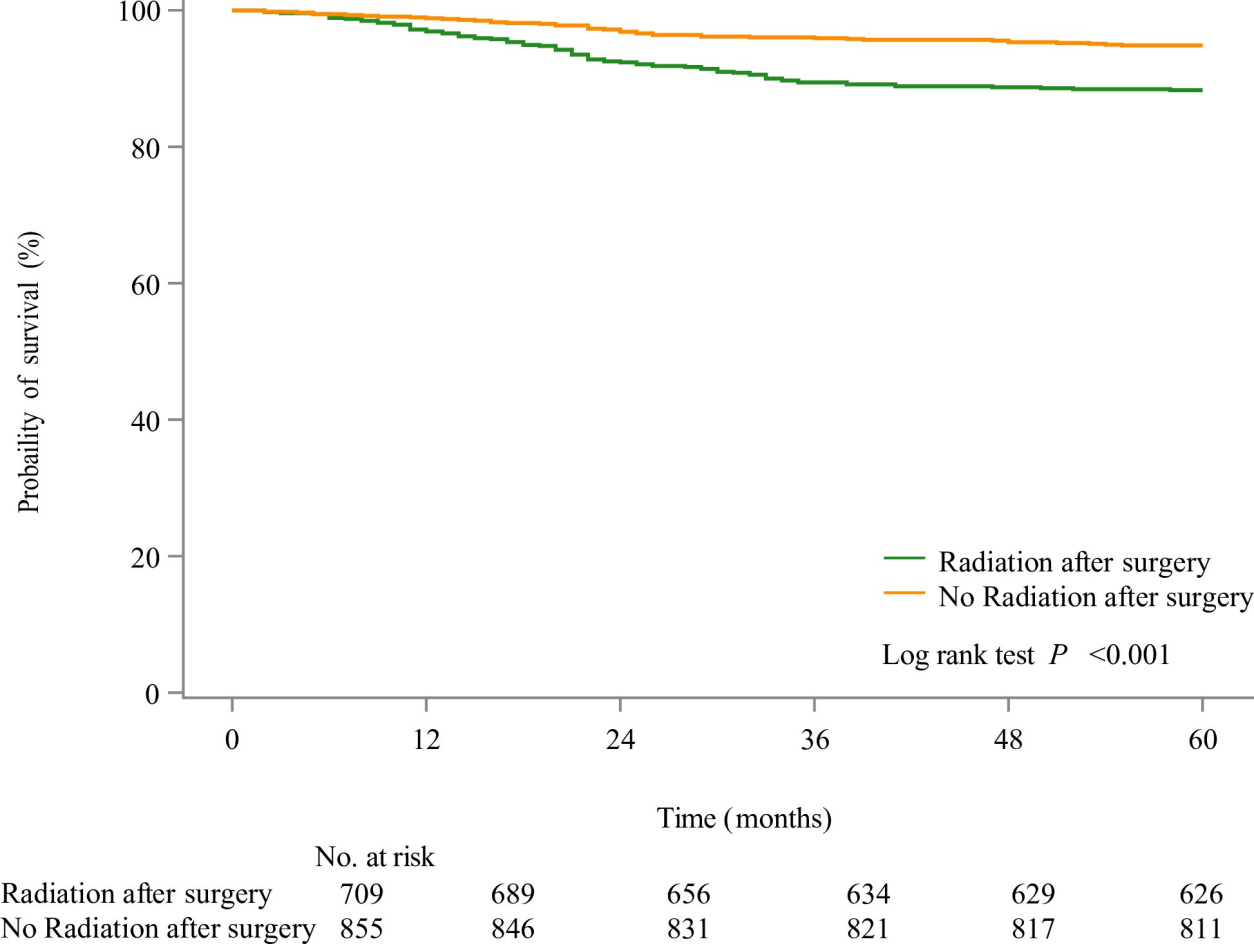
The KM curves of subjects receiving radiation after surgery or not.

**Fig 4:**
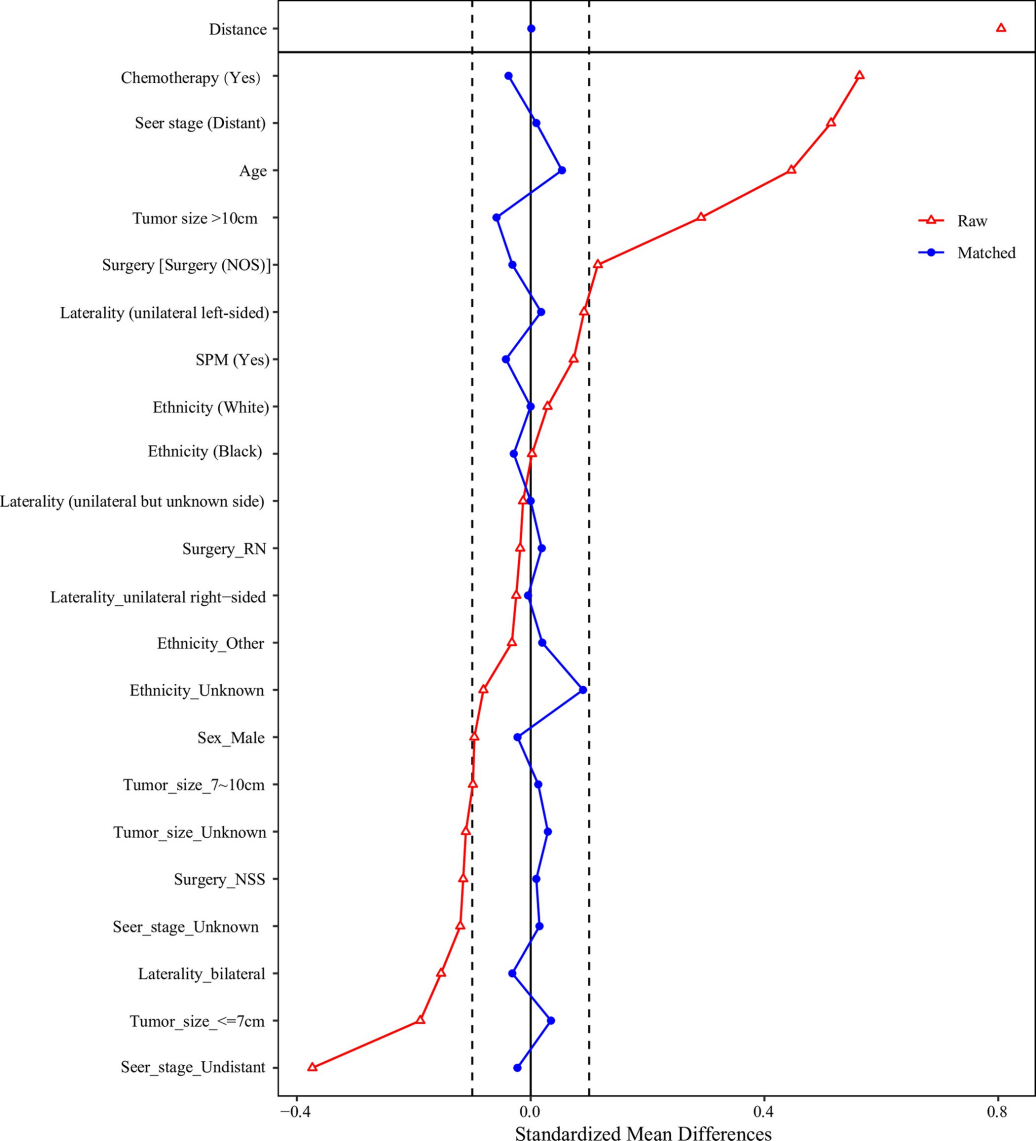
The standardized mean difference of variables before and after PSM.

As presented in Fig 2, we found that patients receiving surgery had higher 5-year survival probability than those not receiving surgery, while participants receiving radiation after surgery showed poor 5-year survival than those not (Fig 3). In the unadjusted model, radiation after surgery might be associated with elevated risk of 5-year overall mortality of patients with Wilms tumor (HR = 2.36, 95%CI: 1.64–3.40). After adjusting for covariates including age and SEER stage, increased risk of 5-year overall mortality in patients with Wilms tumor (HR = 1.62, 95%CI: 1.10–2.41). Radiation after surgery might be a risk factor for 5-year cancer-specific mortality of patients with Wilms tumor (HR = 2.67, 95%CI: 1.81–3.94). After the adjustment for confounding factors including age, SEER stage and ethnicity, increased risk of 5-year cancer-specific mortality of patients with Wilms tumor was observed in those receiving radiation after surgery (HR = 1.77, 95%CI: 1.13–2.79) (Table 3). The baseline characteristics of participant after PSM showed no significant difference between patients receiving radiation after surgery and patients not receiving radiation after surgery (Table 4). The standardized mean difference (SMD) of variables before and after PSM was exhibited in Fig 4. After PSM, we found that radiation after surgery was associated with increased risk of 5-year mortality and 5-year cancer-specific mortality of patients with Wilms tumor (Table 5).

**Table 3:** Associations of radiation after surgery with 5-year OS and 5-year CSS of patients with Wilms tumor.

		Uncorrected		Corrected	
Outcomes	Variables	HR (95%CI)	*P*	HR (95%CI)	*P*
OS	No radiation after surgery	Ref		Ref	
	Radiation after surgery	2.36 (1.64–3.40)	<0.001	1.62 (1.10–2.41)	0.016
CSS	No radiation after surgery	Ref		Ref	
	Radiation after surgery	2.67 (1.81–3.94)	<0.001	1.77 (1.13–2.79)	0.013

HR: Hazard ratio, CI: Confidence interval, OS: Overall survival, CSS: Cancer‐specific survival.

In the adjusted model for OS, age and SEER stage were adjusted.

In the adjusted model for CSS, age, SEER stage and ethnicity were adjusted.

**Table 4:** The characteristics of participants before and after PSM.

		After PSM			
Variables	Total (n = 894)	Radiation after surgery (n = 447)	No radiation after surgery (n = 447)	Statistics	*P*
Age, years, M (Q_1_, Q_3_)	3.00 (2.00, 5.00)	3.00 (1.00, 5.00)	3.00 (2.00, 5.00)	Z = -1.963	0.050
Sex, n (%)				χ^2^ = 0.113	0.737
Female	483 (54.03)	239 (53.47)	244 (54.59)		
Male	411 (45.97)	208 (46.53)	203 (45.41)		
Ethnicity, n (%)				χ^2^ = 4.407	0.221
White	692 (77.40)	346 (77.40)	346 (77.40)		
Black	149 (16.67)	77 (17.23)	72 (16.11)		
Other	50 (5.59)	24 (5.37)	26 (5.82)		
Unknown	3 (0.34)	0 (0.00)	3 (0.67)		
Laterality, n (%)				-	0.958
Unilateral left-sided	412 (46.09)	204 (45.64)	208 (46.53)		
Unilateral right-sided	431 (48.21)	216 (48.32)	215 (48.10)		
Unilateral but unknown side	4 (0.45)	2 (0.45)	2 (0.45)		
Bilateral	47 (5.26)	25 (5.59)	22 (4.92)		
Tumor size, n (%)				χ^2^ = 0.882	0.830
≤7cm	105 (11.74)	50 (11.19)	55 (12.30)		
7-10cm	126 (14.09)	62 (13.87)	64 (14.32)		
>10cm	337 (37.70)	175 (39.15)	162 (36.24)		
Unknown	326 (36.47)	160 (35.79)	166 (37.14)		
Seer stage, n (%)				χ^2^ = 0.121	0.941
Distant	100 (11.19)	49 (10.96)	51 (11.41)		
Non-distant	483 (54.03)	244 (54.59)	239 (53.47)		
Unknown	311 (34.79)	154 (34.45)	157 (35.12)		
Chemotherapy, n (%)				χ^2^ = 0.268	0.605
No/Unknown	35 (3.91)	16 (3.58)	19 (4.25)		
Yes	859 (96.09)	431 (96.42)	428 (95.75)		
Surgery, n (%)				χ^2^ = 0.294	0.863
NSS	63 (7.05)	31 (6.94)	32 (7.16)		
RN	771 (86.24)	384 (85.91)	387 (86.58)		
Surgery (NOS)	60 (6.71)	32 (7.16)	28 (6.26)		
SPM, n (%)				-	0.499
No	892 (99.78)	445 (99.55)	447 (100.00)		
Yes	2 (0.22)	2 (0.45)	0 (0.00)		
5-year mortality, n (%)				χ^2^ = 7.891	0.005
Alive	821 (91.83)	422 (94.41)	399 (89.26)		
Dead	73 (8.17)	25 (5.59)	48 (10.74)		
5-year mortality, n (%)				-	0.006
Alive	821 (91.83)	422 (94.41)	399 (89.26)		
Dead of this cancer	66 (7.38)	21 (4.70)	45 (10.07)		
Dead of other disease	7 (0.78)	4 (0.89)	3 (0.67)		
Survival time, months, M (Q_1_, Q_3_)	133.50 (87.00, 189.00)	141.00 (93.00, 189.00)	123.00 (80.00, 189.00)	Z = 2.296	0.022

NSS: Nephron-sparing surgery, RN: Radical nephrectomy, NOS: Not otherwise specified, SPM: Second primary malignancy; OS: Overall survival, CSS: Cancer-specific survival.

**Table 5:** The associations of radiation after surgery with 5-year OS and 5-year CSS of patients with Wilms tumor before and after PSM.

	Variables	HR (95%CI)	*P*
OS	No radiation after surgery	Ref	
	Radiation after surgery	1.97 (1.22–3.20)	0.006
CSS	No radiation after surgery	Ref	
	Radiation after surgery	2.20 (1.31–3.69)	0.003

HR: Hazard ratio; CI: Confidence interval; OS: Overall survival; CSS: Cancer‐specific survival.

### Subgroup analysis on associations of radiation after surgery with 5-year OS and 5-year CSS of patients with Wilms tumor

Radiation after surgery was associated with elevated risk of 5-year overall mortality in patients aged 4–19 years (HR = 1.83, 95%CI: 1.02–3.28). In females, increased risk of 5-year overall mortality was observed in those receiving radiation after surgery (HR = 2.04, 95%CI: 1.17–3.55). Elevated risk of 5-year overall mortality was identified in those receiving radiation after surgery with unilateral left-sided tumor (HR = 2.02, 95%CI: 1.12–3.64). In patients receiving chemotherapy, radiation after surgery was correlated with increased risk of 5-year overall mortality (HR = 1.65, 95%CI: 1.10–2.48) (Table 6).

**Table 6:** Subgroup analysis on associations of radiation after surgery with 5-year OS and 5-year CSS of patients with Wilms tumor.

	OS		CSS	*P*
Variables	HR (95%CI)	*P*	HR (95%CI)	
Age≤3 years old (n = 963)				
No radiation after surgery	Ref		Ref	
Radiation after surgery	1.41 (0.81–2.46)	0.229	1.67 (0.83–3.37)	0.152
Age in 4~19 years old (n = 601)				
No radiation after surgery	Ref		Ref	
Radiation after surgery	1.83 (1.02–3.28)	0.043	1.75 (0.95–3.22)	0.074
Female (n = 826)				
No radiation after surgery	Ref		Ref	
Radiation after surgery	2.04 (1.17–3.55)	0.012	2.36 (1.24–4.49)	0.009
Male (n = 738)				
No radiation after surgery	Ref		Ref	
Radiation after surgery	1.23 (0.70–2.16)	0.472	1.29 (0.68–2.46)	0.440
Distant Seer stage (n = 282)				
No radiation after surgery	Ref		Ref	
Radiation after surgery	0.75 (0.41–1.38)	0.357	0.71 (0.39–1.30)	0.269
Non-distant Seer stage (n = 815)				
No radiation after surgery	Ref		Ref	
Radiation after surgery	1.83 (0.99–3.39)	0.055	2.11 (1.06–4.19)	0.033
Left side (n = 733)				
No radiation after surgery	Ref		Ref	
Radiation after surgery	2.02 (1.12–3.64)	0.020	2.14 (1.07–4.28)	0.031
Right side (n = 723)				
No radiation after surgery	Ref		Ref	
Radiation after surgery	1.34 (0.74–2.45)	0.338	1.62 (0.83–3.16)	0.160
Bilateral (n = 103)				
No radiation after surgery	Ref		Ref	
Radiation after surgery	2.39 (0.69–8.28)	0.171	2.65 (0.76–9.28)	0.128
Nephron sparing surgery (n = 111)				
No radiation after surgery	Ref		Ref	
Radiation after surgery	5.70 (1.00–32.67)	0.051	5.67 (1.10–29.15)	0.038
Radical nephrectomy (n = 1340)				
No radiation after surgery	Ref		Ref	
Radiation after surgery	1.46 (0.95–2.22)	0.081	1.60 (0.98–2.60)	0.059
No or Unknown Chemotherapy (n = 136)				
No radiation after surgery	Ref		Ref	
Radiation after surgery	0.80 (0.11–5.70)	0.823	0.62 (0.04–10.17)	0.740
Chemotherapy (n = 1428)				
No radiation after surgery	Ref		Ref	
Radiation after surgery	1.65 (1.10–2.48)	0.016	1.85 (1.15–2.95)	0.011
Tumor size: ≤7cm (n = 235)				
No radiation after surgery	Ref		Ref	
Radiation after surgery	1.92 (0.63–5.89)	0.252	2.35 (0.82–6.68)	0.110
Tumor size: 7-10cm (n = 243)				
No radiation after surgery	Ref		Ref	
Radiation after surgery	1.83 (0.56–6.00)	0.321	1.68 (0.41–6.91)	0.471
Tumor size: >10 (n = 584)				
No radiation after surgery	Ref		Ref	
Radiation after surgery	0.99 (0.55–1.77)	0.964	0.97 (0.50–1.89)	0.921
Tumor size: Unknown (n = 502)				
No radiation after surgery	Ref		Ref	
Radiation after surgery	2.81 (1.39–5.69)	0.004	3.47 (1.54–7.82)	0.003

HR: Hazard ratio, CI: Confidence interval, OS: Overall survival, CSS: Cancer‐specific survival.

In the adjusted model for OS, age and SEER stage were adjusted if not stratified.

In the adjusted model for CSS, age, SEER stage and ethnicity were adjusted if not stratified.

The increased risk of 5-year cancer-specific mortality was observed in females receiving radiation after surgery (HR = 2.36, 95%CI: 1.24–4.49). In patients at non-distant SEER stage, radiation after surgery was linked with elevated risk of 5-year cancer-specific mortality (HR = 2.11, 95%CI: 1.06–4.19). Patients receiving radiation after surgery was related with increased risk of 5-year cancer-specific mortality in those with unilateral left-sided tumor (HR = 2.14, 95%CI: 1.07–4.28), those undergoing NSS (HR = 5.67, 95%CI: 1.10–29.15) and those receiving chemotherapy (HR = 1.85, 95%CI: 1.15–2.95) (Table 6).

### Multiple imputation and sensitivity analysis

Missing data of some key variables were dealt with multiple imputation. The results depicted that patients receiving radiation after surgery was correlated with increased the risk of 5-year overall mortality with an adjusted HR of 1.69 (95%CI: 1.15–2.48). The risk of 5-year cancer-specific mortality was increased in those undergoing radiation after surgery (HR = 1.85, 95%CI: 1.20–2.86) (Table 7). Also, Sensitivity analysis was performed to compare the results before and after excluding patients with unknown tumor size, and we found no significant association between radiation after surgery and the risk of 5-year all-cause mortality and 5-year cancer-specific mortality (Table 8).

**Table 7:** Sensitivity analysis comparing the effects of radiation after surgery on 5-year OS and 5-year CSS of patients with Wilms tumor before and after missing value imputation.

Outcomes	Variables	HR (95%CI)	*P*
OS	No radiation after surgery	Ref	
	Radiation after surgery	1.69 (1.15–2.48)	0.008
CSS	No radiation after surgery	Ref	
	Radiation after surgery	1.85 (1.20–2.86)	0.005

HR: Hazard ratio, CI: Confidence interval, OS: Overall survival, CSS: Cancer‐specific survival.

In the adjusted model for OS, age and SEER stage were adjusted.

In the adjusted model for CSS, age, SEER stage and ethnicity were adjusted.

**Table 8:** Sensitivity analysis comparing the effects of radiation after surgery on 5-year OS and 5-year CSS of patients with Wilms tumor before and after deleting tumor size unknown population.

	Variables	HR (95%CI)	*P*
OS	No radiation after surgery	Ref	
	Radiation after surgery	1.24 (0.78–2.00)	0.365
CSS	No radiation after surgery	Ref	
	Radiation after surgery	1.29 (0.75–2.21)	0.361

HR: Hazard ratio, CI: Confidence interval, OS: Overall survival, CSS: Cancer‐specific survival.

In the adjusted model for OS, age and SEER stage were adjusted.

In the adjusted model for CSS, age, and SEER stage were adjusted.

## Discussion

In the present study, the data of 2172 participants diagnosed with Wilms tumor were analyzed to evaluate the association between radiation after surgery and the 5-year OS rate and 5-year CSS rate of these patients. The data delineated that radiation after surgery was associated with increased 5-year overall mortality and 5-year cancer-specific mortality of patients with Wilms tumor. Subgroup analysis showed that radiation after surgery was associated with elevated risk of 5-year overall mortality in patients aged 4–19 years, females, those with unilateral left-sided tumor and patients receiving chemotherapy. Radiation after surgery was related to increased risk of 5-year cancer-specific mortality in females, patients with non-distant SEER stage, unilateral left-sided tumor, undergoing NSS or chemotherapy. The findings of our study might provide a reference for the application of radiation after surgery in Wilms tumor patients.

Radiotherapy is a carcinogenic factor that was reported to be associated with an increased risk of secondary neoplasms in patients especially when those receiving this treatment at young age [15]. Particularly, an elevated risk of secondary cancers has been reported in adult patients treated with radiotherapy for a Wilms tumor in childhood [16–18]. Previously, Sasso et al. found high incidence of late radiation morbidity in patients undergoing adjuvant radiotherapy for Wilms tumor, among them 41% patients suffered from scoliosis 60–180 months from completion of treatment; muscular hypoplasia, length inequality, kyphosis, and iliac wing hypoplasia were seen in 12%, 12%, 15%, and 9% patients, respectively, and the incidence of intestinal occlusion was 20% [19]. The study of Repullo et al. depicted that the risk of hepatocellular carcinoma was increased in those received the combination of surgery and relatively high-dose adjuvant radiotherapy in childhood for Wilms tumor [20]. There evidence gave support to the findings of our study, which delineated that radiation after surgery was associated with increased 5-year overall mortality and 5-year cancer-specific mortality of patients with Wilms tumor. These might due to the complications of radiation in children with Wilms tumor, which suggested the clinicians to caution use radiation after surgery in children with Wilms tumor.

A recent study of Qari et al. explored a case of a three-year-old male with Wilms tumor, and they found that the left and right kidneys respond differently to chemotherapy (the left kidney mass responded poorly to chemotherapy than the right kidney) [21]. The finding indicated that there might be difference between the left kidney and the right kidney. In our study, subgroup analysis depicted that radiation after surgery was associated with increased risk of 5-year mortality in patients with unilateral left-sided tumor. As increasing concerns on preserving renal function and minimizing morbidity of Wilms tumor in children, NSS has been regarded as an alternative therapy [22,23]. This study identified that in patients undergoing NSS, increased risk of 5-year mortality was observed. For patients receiving chemotherapy, radiation after surgery was also associated with elevated risk of 5-year mortality. The findings might suggest that triple therapy might not benefit patients in Wilms tumor, and remind the clinicians to evaluate whether the use of radiation is appropriate in Wilms tumor patients receiving NSS or chemotherapy. There was evidence indicated that after neoadjuvant chemotherapy for Wilms tumor, minimally invasive surgery partial and radical nephrectomies achieved equivalent oncologic fidelity, reduced epidural use and post-operative stays, and better maintained adjuvant therapy timelines when compared to open resections [24]. Ross et al aimed to explore the timing of adjuvant chemotherapy after laparotomy for Wilms tumor and neuroblastoma, although they found that there was no association between early initiation of adjuvant chemotherapy and post-operative complications [25]. These findings suggested in patients receiving radiation after surgery and chemotherapy, the outcomes might vary due to the influence of various factors such as the detailed methods of surgery, and the types of chemotherapy. In female patients who underwent whole abdomen radiotherapy, elevated levels of gonadotropin, primary ovarian failure, one or two small or absent ovaries and a small uterus were observed, suggesting radiation for Wilms tumor might affect other organs of females [26]. In our study, radiation after surgery was associated with increased 5-year overall and cancer-specific mortality in females. Evidence suggested that survivors of childhood cancer have a 6-fold relative risk of developing a secondary malignant neoplasm after high-dose fractionated radiation [27]. Another study revealed that full mantle irradiation was associated with a 2.7-fold increased risk of breast cancer compared with mediastinal irradiation alone in a cohort of 1,112 female 5-year survivors who were treated before the age of 41 years [28]. These findings suggested that the dose and site of radiation might affect the prognosis of patients. Another finding in this study was that no significant association was identified between radiation after surgery and increased risk of 5-year overall mortality and 5-year cancer-specific mortality in patients with distant seer stage. The postoperative treatment, including postoperative radiotherapy for some cancer patients is still controversial. Duranti et al. indicated that radiation therapy was associated with better local control on both univariate and multivariate analysis in patients with thoracic localized soft tissue sarcoma [29]. The findings of another study indicated that the overall survival rates were comparable between patients who underwent surgery with adjuvant radiation and those who only received surgery in an analysis of soft tissue sarcoma patients. It is presumed that the group receiving both radiation therapy and surgery had tumors with more unfavorable characteristics, such as a higher likelihood of close or positive margins, compared to the group treated with surgery alone [30]. Whether radiation after surgery might have value on the in advanced stages of Wilms tumor patients still requires exploration.

This study assessed the association between radiation after surgery and the 5-year OS and 5-year CSS of Wilms tumor patients with a large sample size from the SEER database. The findings might provide a certain reference for the application of radiotherapy in Wilms tumor patients with surgery. Due to the limitation of the SEER database, specific radiotherapy information such as the site and dose could not be obtained. The pathology of tumors [31], and time to the onset of RT after surgery [32] are important factors associated with the prognosis of patients with cancers. The presence of residual disease after surgery is associated with the risk of recurrence [33], The area and dose of RT were widely accepted to affect the prognosis of cancer patients [34]. However, these data were not reported in the SEER database. The International Society of Paediatric Oncology (SIOP) and Children’s oncology group (COG) staging for Wilms tumor are important for evaluating the prognosis and treatment strategies of these patients, which were not recorded and evaluated by SEER. The SEER stage of WT is not sufficient today for patients’ risk stratification. Also, the sample size of unknown population of tumor size was big, and the results from sensitivity analysis indicated that patients with unknown tumor size should be included, and excluded this population might cause bias. There were 91.3% of patients received chemotherapy, and chemotherapy at least includes neoadjuvant chemotherapy and adjuvant chemotherapy, but the detailed types and purposes for these patients were unclear according to the data in the database, which might affect the results of our study. In addition, the incidence of SPM was low in our study, whether there was association between radiation after surgery and the occurrence of SPM could not be analyzed. In the future, more well-designed studies were required to verify the findings in our study.

## Conclusions

The current study evaluated the association between radiation after surgery and the 5-year OS rate and 5-year CSS rate of patients with Wilms tumor based on the data of 1564 participants. The findings of our study revealed that radiation after surgery was associated with poor prognosis of patients with Wilms tumor. These results indicated that the clinicians should assess whether the patient was suitable for using radiation after surgery.

## References

[1] Leslie SW, Sajjad H, Murphy PB. Wilms Tumor. StatPearls. Treasure Island (FL): StatPearls Publishing Copyright © 2022, StatPearls Publishing LLC.; 2022.

[2] Uittenboogaard A, Njuguna F, Mostert S, Langat S, van de Velde ME, Olbara G, et al. Outcomes of Wilms tumor treatment in western Kenya. Pediatric blood & cancer. 2022;69(4):e29503. Epub 2021/12/16. doi: 10.1002/pbc.29503 . 34908225

[3] Awal HB, Nandula SR, Domingues CC, Dore FJ, Kundu N, Brichacek B, et al. Linagliptin, when compared to placebo, improves CD34+ve endothelial progenitor cells in type 2 diabetes subjects with chronic kidney disease taking metformin and/or insulin: a randomized controlled trial. Cardiovascular diabetology. 2020;19(1):72. Epub 2020/06/05. doi: 10.1186/s12933-020-01046-z ; PubMed Central PMCID: PMC7271387. 32493344 PMC7271387

[4] Koshinaga T, Takimoto T, Oue T, Okita H, Tanaka Y, Nozaki M, et al. Outcome of renal tumors registered in Japan Wilms Tumor Study-2 (JWiTS-2): A report from the Japan Children’s Cancer Group (JCCG). Pediatric blood & cancer. 2018;65(7):e27056. Epub 2018/04/10. doi: 10.1002/pbc.27056 . 29630767

[5] Israels T, Paintsil V, Nyirenda D, Kouya F, Mbah Afungchwi G, Hesseling P, et al. Improved outcome at end of treatment in the collaborative Wilms tumour Africa project. Pediatric blood & cancer. 2018;65(5):e26945. Epub 2018/01/20. doi: 10.1002/pbc.26945 . 29350457

[6] Joko-Fru WY, Parkin DM, Borok M, Chokunonga E, Korir A, Nambooze S, et al. Survival from childhood cancers in Eastern Africa: A population-based registry study. International journal of cancer. 2018;143(10):2409–15. Epub 2018/07/08. doi: 10.1002/ijc.31723 . 29981149

[7] Imam N, Burjonrappa S. Nephron sparing surgery outcomes in Wilms’ tumor: is it ready for primetime? Pediatric surgery international. 2022;39(1):5. Epub 2022/11/29. doi: 10.1007/s00383-022-05299-5 . 36441254

[8] Balis F, Green DM, Anderson C, Cook S, Dhillon J, Gow K, et al. Wilms Tumor (Nephroblastoma), Version 2.2021, NCCN Clinical Practice Guidelines in Oncology. Journal of the National Comprehensive Cancer Network: JNCCN. 2021;19(8):945–77. Epub 2021/08/21. doi: 10.6004/jnccn.2021.0037 34416707

[9] Jairam V, Roberts KB, Yu JB. Historical trends in the use of radiation therapy for pediatric cancers: 1973–2008. International journal of radiation oncology, biology, physics. 2013;85(3):e151–5. Epub 2013/01/01. doi: 10.1016/j.ijrobp.2012.10.007 ; PubMed Central PMCID: PMC3636568. 23273995 PMC3636568

[10] De Ruysscher D, Niedermann G, Burnet NG, Siva S, Lee AWM, Hegi-Johnson F. Radiotherapy toxicity. Nature reviews Disease primers. 2019;5(1):13. Epub 2019/02/23. doi: 10.1038/s41572-019-0064-5 . 30792503

[11] Kamran SC, Berrington de Gonzalez A, Ng A, Haas-Kogan D, Viswanathan AN. Therapeutic radiation and the potential risk of second malignancies. Cancer. 2016;122(12):1809–21. Epub 2016/03/08. doi: 10.1002/cncr.29841 . 26950597

[12] Friedman DL, Whitton J, Leisenring W, Mertens AC, Hammond S, Stovall M, et al. Subsequent neoplasms in 5-year survivors of childhood cancer: the Childhood Cancer Survivor Study. Journal of the National Cancer Institute. 2010;102(14):1083–95. Epub 2010/07/17. doi: 10.1093/jnci/djq238 ; PubMed Central PMCID: PMC2907408. 20634481 PMC2907408

[13] Bhambhvani HP, Peterson DJ, Sheth KR. Sociodemographic factors associated with Wilms tumor treatment and survival: a population-based study. International urology and nephrology. 2022. Epub 2022/09/08. doi: 10.1007/s11255-022-03343-w . 36069962

[14] Chen L, Ma J, Zou Z, Liu H, Liu C, Gong S, et al. Clinical characteristics and prognosis of patients with glioblastoma: A review of survival analysis of 1674 patients based on SEER database. Medicine. 2022;101(47):e32042. Epub 2022/12/02. doi: 10.1097/MD.0000000000032042 ; PubMed Central PMCID: PMC9704894. 36451503 PMC9704894

[15] Berrington de Gonzalez A, Curtis RE, Kry SF, Gilbert E, Lamart S, Berg CD, et al. Proportion of second cancers attributable to radiotherapy treatment in adults: a cohort study in the US SEER cancer registries. The Lancet Oncology. 2011;12(4):353–60. Epub 2011/04/02. doi: 10.1016/S1470-2045(11)70061-4 ; PubMed Central PMCID: PMC3086738. 21454129 PMC3086738

[16] Wong KF, Reulen RC, Winter DL, Guha J, Fidler MM, Kelly J, et al. Risk of Adverse Health and Social Outcomes Up to 50 Years After Wilms Tumor: The British Childhood Cancer Survivor Study. Journal of clinical oncology: official journal of the American Society of Clinical Oncology. 2016;34(15):1772–9. Epub 2016/03/30. doi: 10.1200/JCO.2015.64.4344 . 27022116

[17] Breslow NE, Norkool PA, Olshan A, Evans A, D’Angio GJ. Second malignant neoplasms in survivors of Wilms’ tumor: a report from the National Wilms’ Tumor Study. Journal of the National Cancer Institute. 1988;80(8):592–5. Epub 1988/06/15. doi: 10.1093/jnci/80.8.592 . 2836600

[18] Li FP, Yan JC, Sallan S, Cassady JR, Jr., Danahy J, Fine W, et al. Second neoplasms after Wilms’ tumor in childhood. Journal of the National Cancer Institute. 1983;71(6):1205–9. Epub 1983/12/01. . 6317934

[19] Sasso G, Greco N, Murino P, Sasso FS. Late toxicity in Wilms tumor patients treated with radiotherapy at 15 years of median follow-up. Journal of pediatric hematology/oncology. 2010;32(7):e264–7. Epub 2010/08/26. doi: 10.1097/MPH.0b013e3181e7931a . 20736847

[20] Repullo D, Diaz M, Holbrechts S, Gomez-Galdón M, Van Gestel D, Bohlok A, et al. Unusual presentation of a hepatocellular carcinoma as a potential late side effect of radiotherapy in a patient treated for Wilms tumor in childhood. World journal of surgical oncology. 2018;16(1):48. Epub 2018/03/09. doi: 10.1186/s12957-018-1346-1 ; PubMed Central PMCID: PMC5842595. 29514643 PMC5842595

[21] Qari W, Alzahrani A, Alzahrani M, Saleh Y, Almasabi A, Bawazir O. Bilateral Wilms’ Tumor With Different Responses to Preoperative Chemotherapy. Cureus. 2022;14(10):e30593. Epub 2022/11/26. doi: 10.7759/cureus.30593 ; PubMed Central PMCID: PMC9681715. 36426331 PMC9681715

[22] Mrad C, Audry G, Tabone MD, Le Pointe HD, Coulomb A, Irtan S. Nephron Sparing Surgery in Bilateral Wilms Tumors With Botryoid Growth Pattern. Journal of pediatric hematology/oncology. 2022;44(3):e740–e2. Epub 2021/09/26. doi: 10.1097/MPH.0000000000002337 . 34561400

[23] Fang Y, Li Z, Song H, Sun N, Zhang W. Treatment of bilateral Wilms’ tumor in children: how to improve the application of nephron-sparing surgery. Pediatric surgery international. 2023;39(1):145. Epub 2023/03/02. doi: 10.1007/s00383-023-05433-x . 36856873

[24] McKay KG, Abdul Ghani MO, Crane GL, Evans PT, Zhao S, Martin LY, et al. Oncologic Fidelity of Minimally Invasive Surgery to Resect Neoadjuvant-Treated Wilms Tumors. Am Surg. 2022;88(5):943–52. Epub 2022/01/08. doi: 10.1177/00031348211070796 . 34994212

[25] Ross A, Gomez O, Wang X, Lu Z, Abdelhafeez H, Davidoff AM, et al. Timing of adjuvant chemotherapy after laparotomy for Wilms tumor and neuroblastoma. Pediatr Surg Int. 2021;37(11):1585–92. Epub 2021/07/17. doi: 10.1007/s00383-021-04968-1 ; PubMed Central PMCID: PMC8530895. 34268609 PMC8530895

[26] Nussbaum Blask AR, Nicholson HS, Markle BM, Wechsler-Jentzch K, O’Donnell R, Byrne J. Sonographic detection of uterine and ovarian abnormalities in female survivors of Wilms’ tumor treated with radiotherapy. AJR Am J Roentgenol. 1999;172(3):759–63. Epub 1999/03/04. doi: 10.2214/ajr.172.3.10063876 . 10063876

[27] Kamran SC, Berrington de Gonzalez A, Ng A, Haas-Kogan D, Viswanathan AN. Therapeutic radiation and the potential risk of second malignancies. Cancer. 2016;122(12):1809–21. Epub 2016/03/08. doi: 10.1002/cncr.29841 . 26950597

[28] De Bruin ML, Sparidans J, van’t Veer MB, Noordijk EM, Louwman MW, Zijlstra JM, et al. Breast cancer risk in female survivors of Hodgkin’s lymphoma: lower risk after smaller radiation volumes. Journal of clinical oncology: official journal of the American Society of Clinical Oncology. 2009;27(26):4239–46. Epub 2009/08/12. doi: 10.1200/JCO.2008.19.9174 . 19667275

[29] Duranti L, Gronchi A, Stacchiotti S, Fiore M, Casali PG, Collini P, et al. Localised thoracic sarcomas: outcome improvement over time at a single institution. European journal of cancer (Oxford, England: 1990). 2013;49(12):2689–97. Epub 2013/05/21. doi: 10.1016/j.ejca.2013.04.007 . 23683891

[30] Wushou A, Jiang YZ, Liu YR, Shao ZM. The demographic features, clinicopathologic characteristics, treatment outcome and disease-specific prognostic factors of solitary fibrous tumor: a population-based analysis. Oncotarget. 2015;6(39):41875–83. Epub 2015/10/27. doi: 10.18632/oncotarget.6174 ; PubMed Central PMCID: PMC4747195. 26496033 PMC4747195

[31] Zhang Y, Wu Q, Warrick JI, DeGraff DJ, Raman JD, Truong H, et al. Clinicopathological risk factors associated with tumor relapse of upper tract urothelial carcinoma after radical nephroureterectomy: A single institution 20-year experience. Annals of diagnostic pathology. 2024;73:152357. Epub 2024/06/29. doi: 10.1016/j.anndiagpath.2024.152357 . 38941945

[32] Hara J, Nitani C, Shichino H, Kuroda T, Hishiki T, Soejima T, et al. Outcome of children with relapsed high-risk neuroblastoma in Japan and analysis of the role of allogeneic hematopoietic stem cell transplantation. Japanese journal of clinical oncology. 2022;52(5):486–92. Epub 2022/02/10. doi: 10.1093/jjco/hyac007 . 35137156

[33] Oh HH, Kim JS, Lim JW, Lim CJ, Seo YE, You GR, et al. Clinical outcomes of colorectal neoplasm with positive resection margin after endoscopic submucosal dissection. Scientific reports. 2024;14(1):12353. Epub 2024/05/30. doi: 10.1038/s41598-024-63129-1 ; PubMed Central PMCID: PMC11136969. 38811758 PMC11136969

[34] Chen Y, Li WX, Wu JH, Chen GH, Yang CM, Lu H, et al. Does the Dose of Standard Adjuvant Chemotherapy Affect the Triple-negative Breast Cancer Benefit from Extended Capecitabine Metronomic Therapy? An Exploratory Analysis of the SYSUCC-001 Trial. Breast cancer (Dove Medical Press). 2024;16:223–31. Epub 2024/04/17. doi: 10.2147/BCTT.S447290 ; PubMed Central PMCID: PMC11020346. 38628818 PMC11020346

